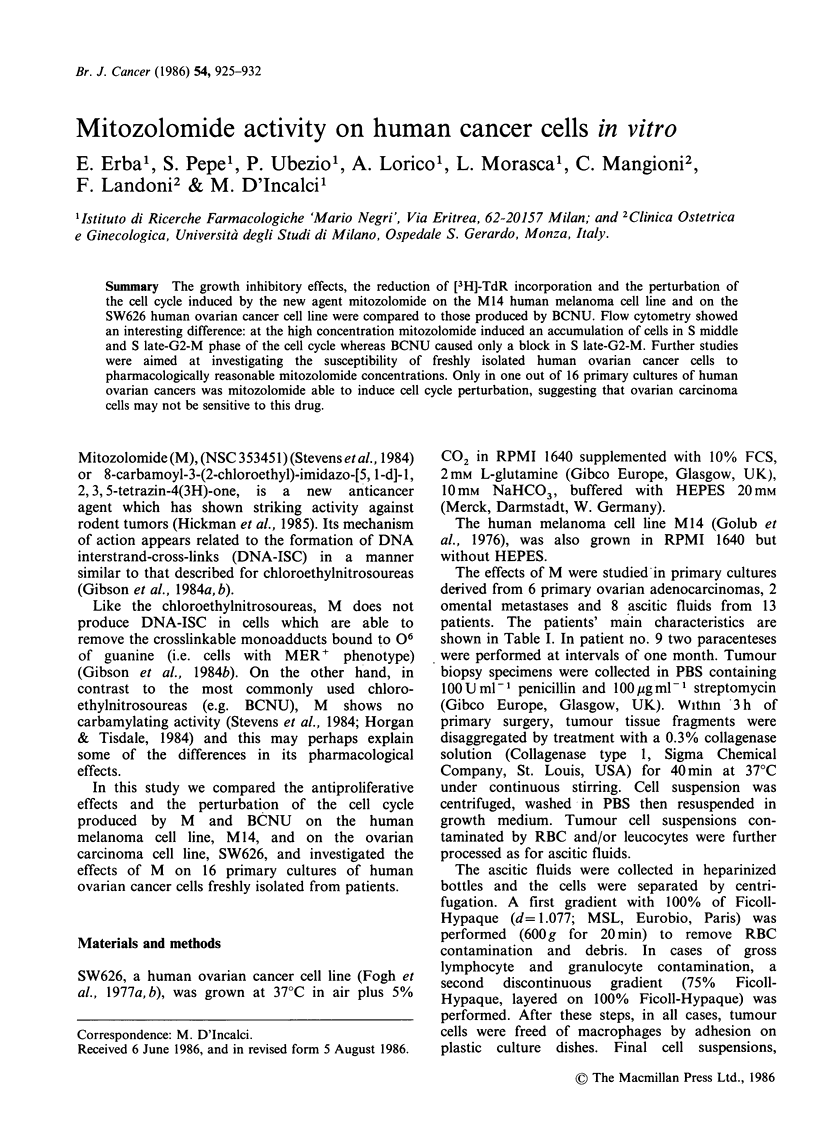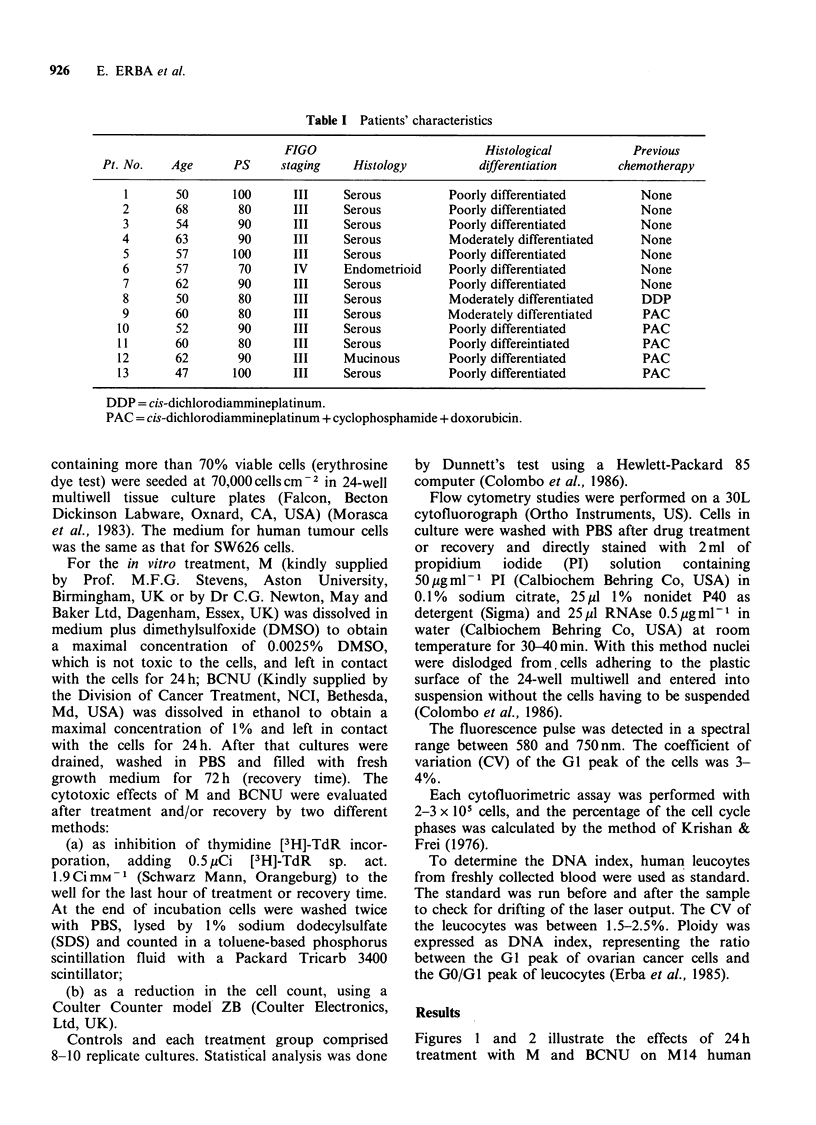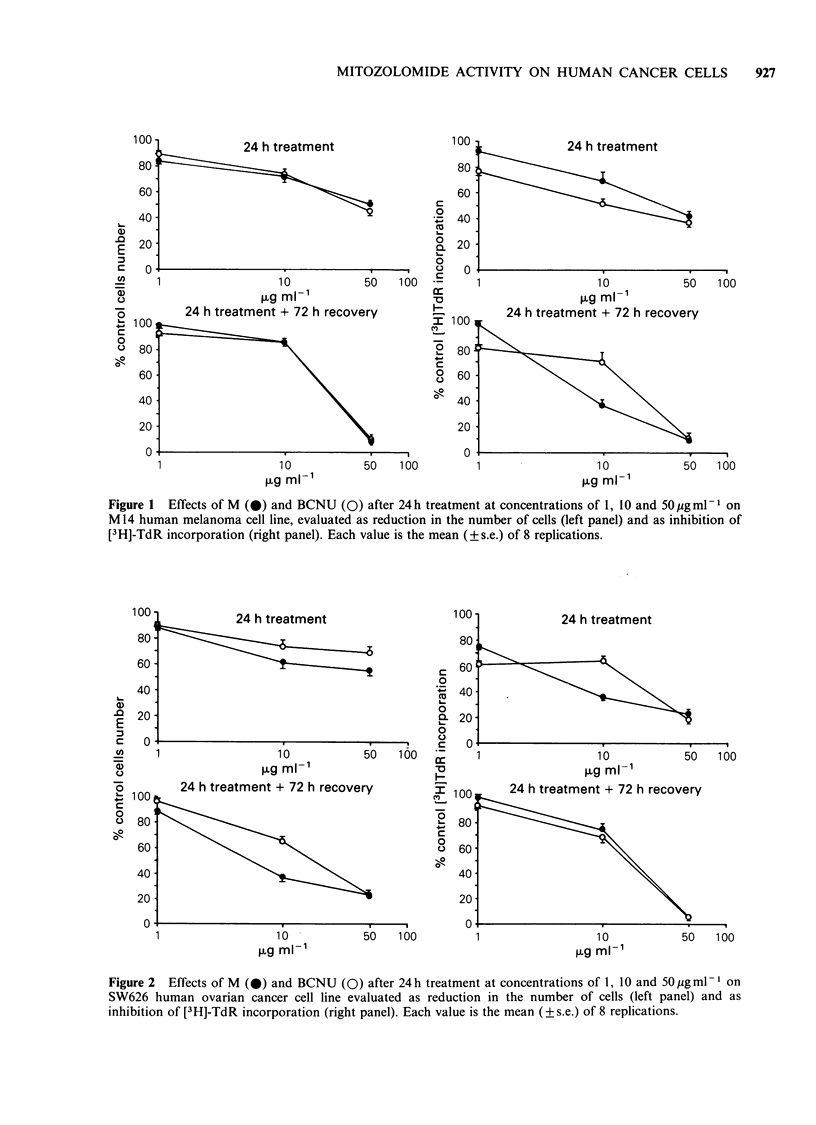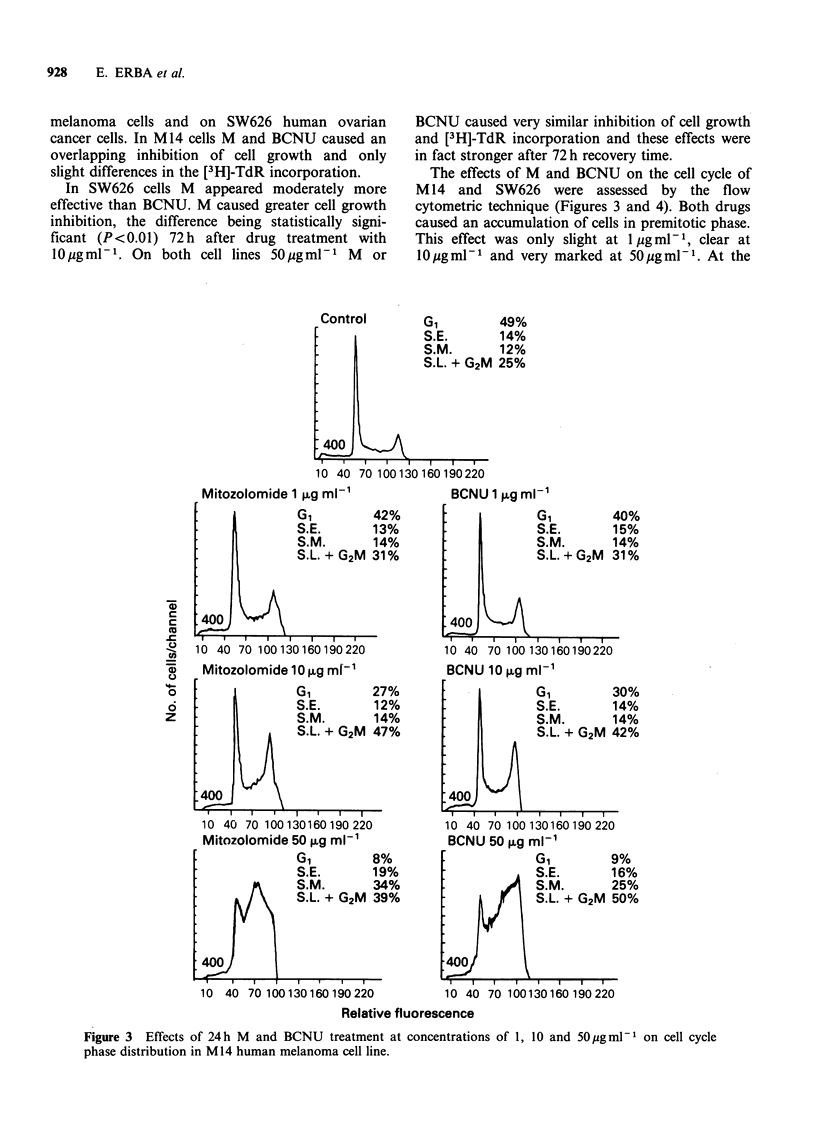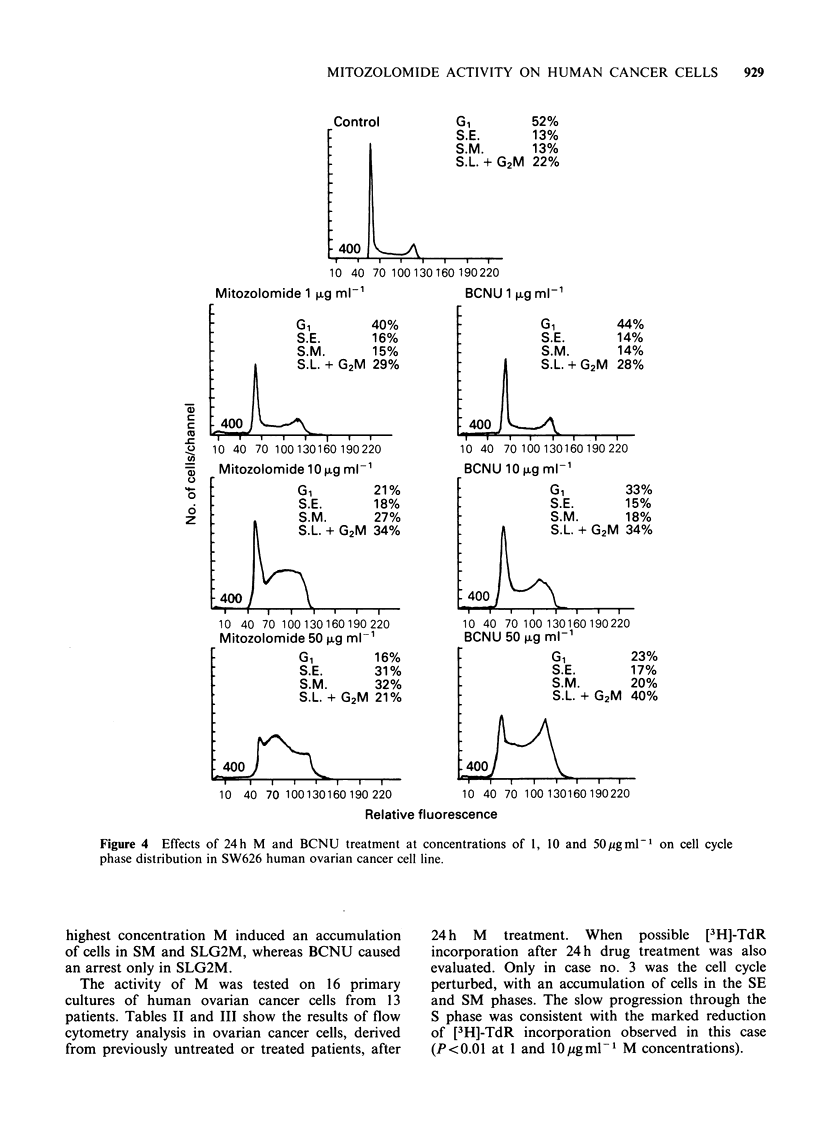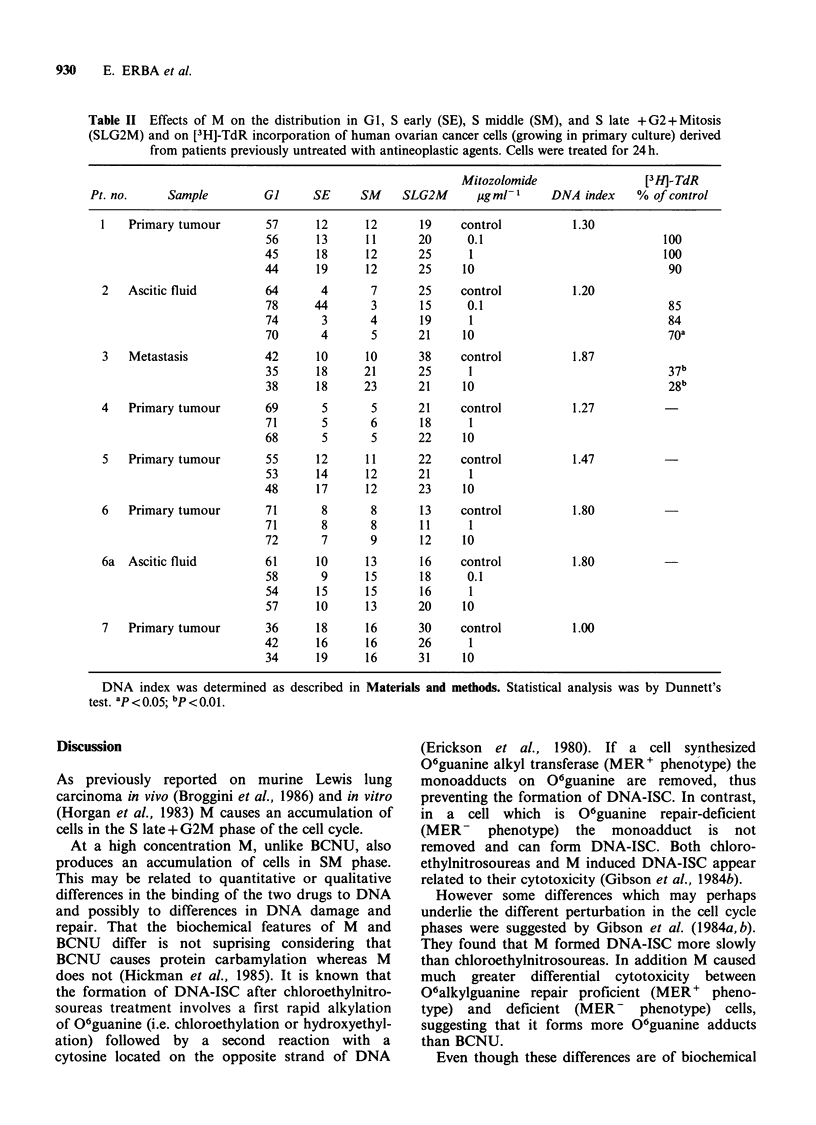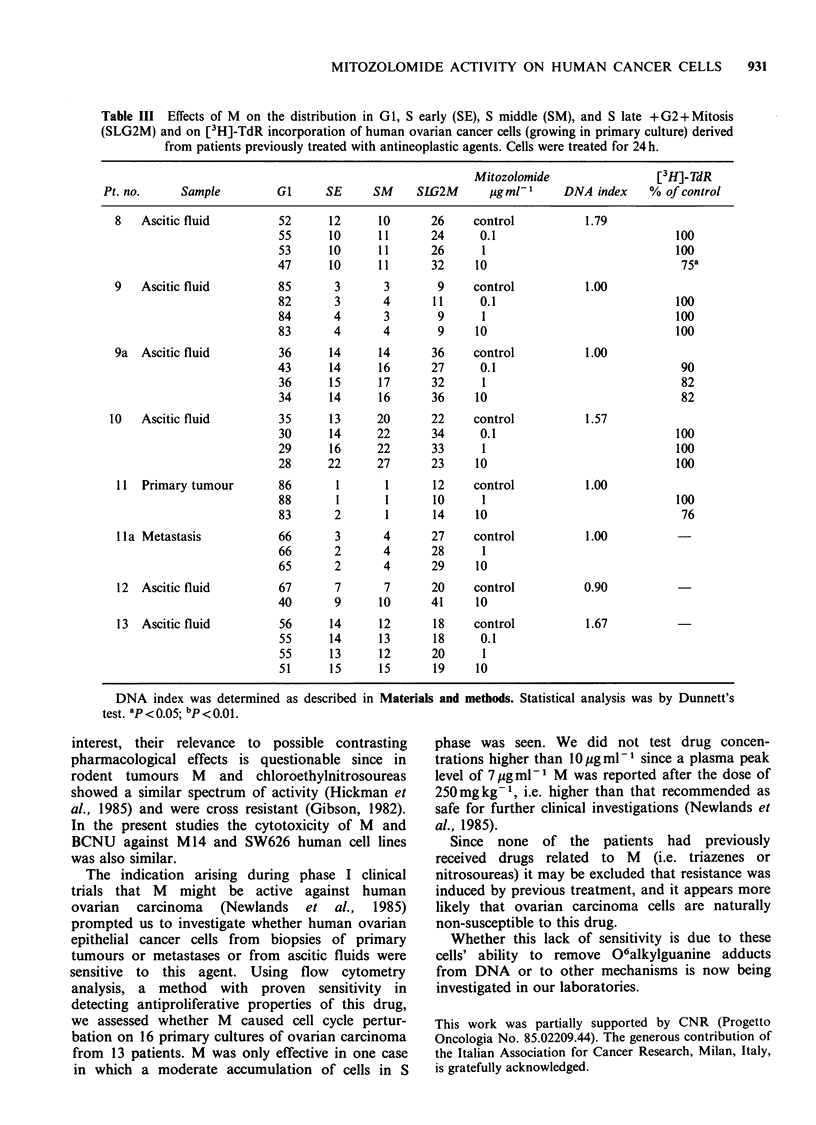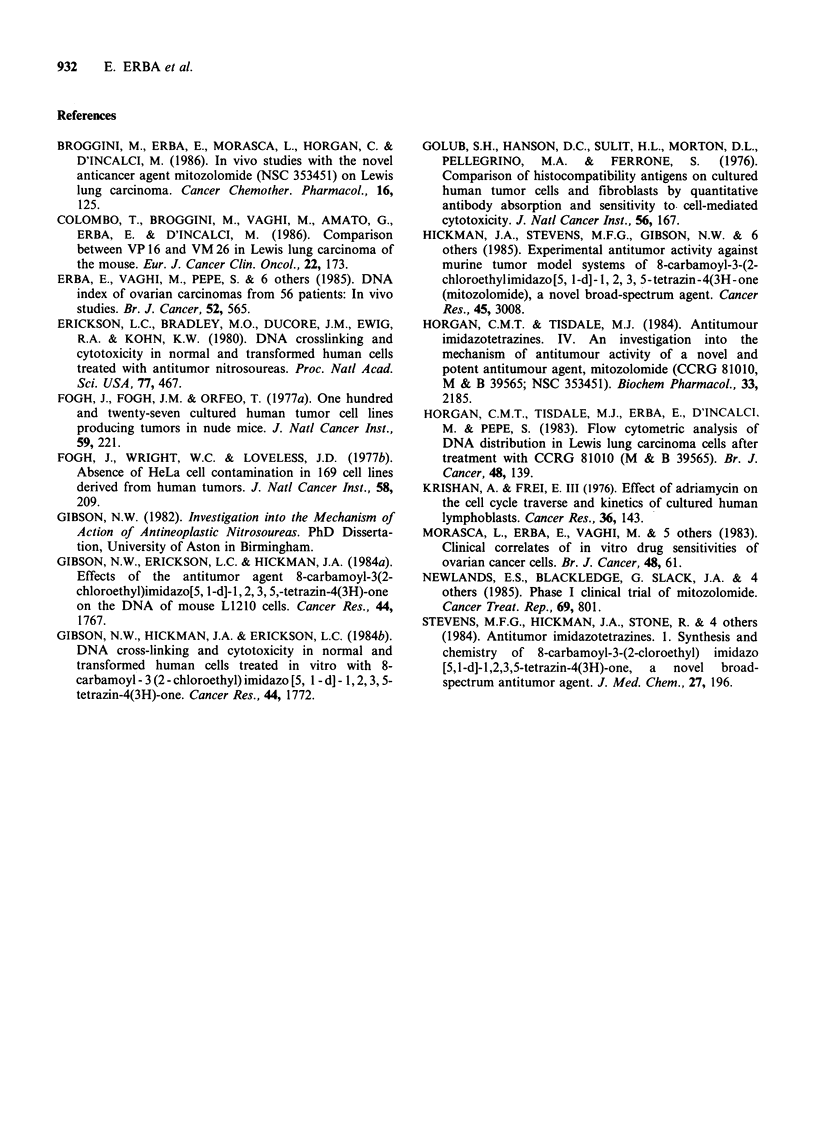# Mitozolomide activity on human cancer cells in vitro.

**DOI:** 10.1038/bjc.1986.263

**Published:** 1986-12

**Authors:** E. Erba, S. Pepe, P. Ubezio, A. Lorico, L. Morasca, C. Mangioni, F. Landoni, M. D'Incalci

## Abstract

The growth inhibitory effects, the reduction of [3H]-TdR incorporation and the perturbation of the cell cycle induced by the new agent mitozolomide on the M14 human melanoma cell line and on the SW626 human ovarian cancer cell line were compared to those produced by BCNU. Flow cytometry showed an interesting difference: at the high concentration mitozolomide induced an accumulation of cells in S middle and S late-G2-M phase of the cell cycle whereas BCNU caused only a block in S late-G2-M. Further studies were aimed at investigating the susceptibility of freshly isolated human ovarian cancer cells to pharmacologically reasonable mitozolomide concentrations. Only in one out of 16 primary cultures of human ovarian cancers was mitozolomide able to induce cell cycle perturbation, suggesting that ovarian carcinoma cells may not be sensitive to this drug.


Br. J. Cancer (1986) **54**, 925–932

### Mitozolomide activity on human cancer cells *in vitro*

E. Erba[1], S. Pepe[1], P. Ubezio[1], A. Lorico[1], L. Morasca[1], C. Mangioni[2], F. Landoni[2] & M. D'Incalci[1]

[1]*Istituto di Ricerche Farmacologiche 'Mario Negri', Via Eritrea, 62-20157 Milan; and* [2]*Clinica Ostetrica e Ginecologica, Università degli Studi di Milano, Ospedale S. Gerardo, Monza, Italy.*

**Summary** The growth inhibitory effects, the reduction of [$^3$H]-TdR incorporation and the perturbation of the cell cycle induced by the new agent mitozolomide on the M14 human melanoma cell line and on the SW626 human ovarian cancer cell line were compared to those produced by BCNU. Flow cytometry showed an interesting difference: at the high concentration mitozolomide induced an accumulation of cells in S middle and S late-G2-M phase of the cell cycle whereas BCNU caused only a block in S late-G2-M. Further studies were aimed at investigating the susceptibility of freshly isolated human ovarian cancer cells to pharmacologically reasonable mitozolomide concentrations. Only in one out of 16 primary cultures of human ovarian cancers was mitozolomide able to induce cell cycle perturbation, suggesting that ovarian carcinoma cells may not be sensitive to this drug.

Mitozolomide (M), (NSC 353451) (Stevens *et al.*, 1984) or 8-carbamoyl-3-(2-chloroethyl)-imidazo-[5, 1-d]-1, 2, 3, 5-tetrazin-4(3H)-one, is a new anticancer agent which has shown striking activity against rodent tumors (Hickman *et al.*, 1985). Its mechanism of action appears related to the formation of DNA interstrand-cross-links (DNA-ISC) in a manner similar to that described for chloroethylnitrosoureas (Gibson *et al.*, 1984*a, b*).

Like the chloroethylnitrosoureas, M does not produce DNA-ISC in cells which are able to remove the crosslinkable monoadducts bound to $O^6$ of guanine (i.e. cells with $MER^+$ phenotype) (Gibson *et al.*, 1984*b*). On the other hand, in contrast to the most commonly used chloroethylnitrosoureas (e.g. BCNU), M shows no carbamylating activity (Stevens *et al.*, 1984; Horgan & Tisdale, 1984) and this may perhaps explain some of the differences in its pharmacological effects.

In this study we compared the antiproliferative effects and the perturbation of the cell cycle produced by M and BCNU on the human melanoma cell line, M14, and on the ovarian carcinoma cell line, SW626, and investigated the effects of M on 16 primary cultures of human ovarian cancer cells freshly isolated from patients.

### Materials and methods

SW626, a human ovarian cancer cell line (Fogh *et al.*, 1977*a, b*), was grown at 37°C in air plus 5% $CO_2$ in RPMI 1640 supplemented with 10% FCS, 2 mM L-glutamine (Gibco Europe, Glasgow, UK), 10 mM $NaHCO_3$, buffered with HEPES 20 mM (Merck, Darmstadt, W. Germany).

The human melanoma cell line M14 (Golub *et al.*, 1976), was also grown in RPMI 1640 but without HEPES.

The effects of M were studied in primary cultures derived from 6 primary ovarian adenocarcinomas, 2 omental metastases and 8 ascitic fluids from 13 patients. The patients' main characteristics are shown in Table I. In patient no. 9 two paracenteses were performed at intervals of one month. Tumour biopsy specimens were collected in PBS containing 100 U ml$^{-1}$ penicillin and 100 $\mu$g ml$^{-1}$ streptomycin (Gibco Europe, Glasgow, UK). Within 3 h of primary surgery, tumour tissue fragments were disaggregated by treatment with a 0.3% collagenase solution (Collagenase type 1, Sigma Chemical Company, St. Louis, USA) for 40 min at 37°C under continuous stirring. Cell suspension was centrifuged, washed in PBS then resuspended in growth medium. Tumour cell suspensions contaminated by RBC and/or leucocytes were further processed as for ascitic fluids.

The ascitic fluids were collected in heparinized bottles and the cells were separated by centrifugation. A first gradient with 100% of Ficoll-Hypaque ($d$ = 1.077; MSL, Eurobio, Paris) was performed (600 $g$ for 20 min) to remove RBC contamination and debris. In cases of gross lymphocyte and granulocyte contamination, a second discontinuous gradient (75% Ficoll-Hypaque, layered on 100% Ficoll-Hypaque) was performed. After these steps, in all cases, tumour cells were freed of macrophages by adhesion on plastic culture dishes. Final cell suspensions,

Correspondence: M. D'Incalci.
Received 6 June 1986, and in revised form 5 August 1986.

© The Macmillan Press Ltd., 1986

**Table I** Patients' characteristics

| *Pt. No.* | *Age* | *PS* | *FIGO staging* | *Histology* | *Histological differentiation* | *Previous chemotherapy* |
|---|---|---|---|---|---|---|
| 1 | 50 | 100 | III | Serous | Poorly differentiated | None |
| 2 | 68 | 80 | III | Serous | Poorly differentiated | None |
| 3 | 54 | 90 | III | Serous | Poorly differentiated | None |
| 4 | 63 | 90 | III | Serous | Moderately differentiated | None |
| 5 | 57 | 100 | III | Serous | Poorly differentiated | None |
| 6 | 57 | 70 | IV | Endometrioid | Poorly differentiated | None |
| 7 | 62 | 90 | III | Serous | Poorly differentiated | None |
| 8 | 50 | 80 | III | Serous | Moderately differentiated | DDP |
| 9 | 60 | 80 | III | Serous | Moderately differentiated | PAC |
| 10 | 52 | 90 | III | Serous | Poorly differentiated | PAC |
| 11 | 60 | 80 | III | Serous | Poorly differeintiated | PAC |
| 12 | 62 | 90 | III | Mucinous | Poorly differentiated | PAC |
| 13 | 47 | 100 | III | Serous | Poorly differentiated | PAC |

DDP = *cis*-dichlorodiammineplatinum.
PAC = *cis*-dichlorodiammineplatinum + cyclophosphamide + doxorubicin.

containing more than 70% viable cells (erythrosine dye test) were seeded at 70,000 cells cm$^{-2}$ in 24-well multiwell tissue culture plates (Falcon, Becton Dickinson Labware, Oxnard, CA, USA) (Morasca *et al.*, 1983). The medium for human tumour cells was the same as that for SW626 cells.

For the *in vitro* treatment, M (kindly supplied by Prof. M.F.G. Stevens, Aston University, Birmingham, UK or by Dr C.G. Newton, May and Baker Ltd, Dagenham, Essex, UK) was dissolved in medium plus dimethylsulfoxide (DMSO) to obtain a maximal concentration of 0.0025% DMSO, which is not toxic to the cells, and left in contact with the cells for 24 h; BCNU (Kindly supplied by the Division of Cancer Treatment, NCI, Bethesda, Md, USA) was dissolved in ethanol to obtain a maximal concentration of 1% and left in contact with the cells for 24 h. After that cultures were drained, washed in PBS and filled with fresh growth medium for 72 h (recovery time). The cytotoxic effects of M and BCNU were evaluated after treatment and/or recovery by two different methods:

(a) as inhibition of thymidine [$^3$H]-TdR incorporation, adding 0.5 $\mu$Ci [$^3$H]-TdR sp. act. 1.9 Ci mM$^{-1}$ (Schwarz Mann, Orangeburg) to the well for the last hour of treatment or recovery time. At the end of incubation cells were washed twice with PBS, lysed by 1% sodium dodecylsulfate (SDS) and counted in a toluene-based phosphorus scintillation fluid with a Packard Tricarb 3400 scintillator;

(b) as a reduction in the cell count, using a Coulter Counter model ZB (Coulter Electronics, Ltd, UK).

Controls and each treatment group comprised 8–10 replicate cultures. Statistical analysis was done by Dunnett's test using a Hewlett-Packard 85 computer (Colombo *et al.*, 1986).

Flow cytometry studies were performed on a 30L cytofluorograph (Ortho Instruments, US). Cells in culture were washed with PBS after drug treatment or recovery and directly stained with 2 ml of propidium iodide (PI) solution containing 50 $\mu$g ml$^{-1}$ PI (Calbiochem Behring Co, USA) in 0.1% sodium citrate, 25 $\mu$l 1% nonidet P40 as detergent (Sigma) and 25 $\mu$l RNAse 0.5 $\mu$g ml$^{-1}$ in water (Calbiochem Behring Co, USA) at room temperature for 30–40 min. With this method nuclei were dislodged from cells adhering to the plastic surface of the 24-well multiwell and entered into suspension without the cells having to be suspended (Colombo *et al.*, 1986).

The fluorescence pulse was detected in a spectral range between 580 and 750 nm. The coefficient of variation (CV) of the G1 peak of the cells was 3–4%.

Each cytofluorimetric assay was performed with 2–3 x 10$^5$ cells, and the percentage of the cell cycle phases was calculated by the method of Krishan & Frei (1976).

To determine the DNA index, human leucoytes from freshly collected blood were used as standard. The standard was run before and after the sample to check for drifting of the laser output. The CV of the leucocytes was between 1.5–2.5%. Ploidy was expressed as DNA index, representing the ratio between the G1 peak of ovarian cancer cells and the G0/G1 peak of leucocytes (Erba *et al.*, 1985).

### Results

Figures 1 and 2 illustrate the effects of 24 h treatment with M and BCNU on M14 human

**Figure 1** Effects of M (●) and BCNU (○) after 24 h treatment at concentrations of 1, 10 and 50 $\mu g\,ml^{-1}$ on M14 human melanoma cell line, evaluated as reduction in the number of cells (left panel) and as inhibition of [$^3$H]-TdR incorporation (right panel). Each value is the mean (±s.e.) of 8 replications.

**Figure 2** Effects of M (●) and BCNU (○) after 24 h treatment at concentrations of 1, 10 and 50 $\mu g\,ml^{-1}$ on SW626 human ovarian cancer cell line evaluated as reduction in the number of cells (left panel) and as inhibition of [$^3$H]-TdR incorporation (right panel). Each value is the mean (±s.e.) of 8 replications.

melanoma cells and on SW626 human ovarian cancer cells. In M14 cells M and BCNU caused an overlapping inhibition of cell growth and only slight differences in the [$^3$H]-TdR incorporation.

In SW626 cells M appeared moderately more effective than BCNU. M caused greater cell growth inhibition, the difference being statistically significant ($P<0.01$) 72 h after drug treatment with $10\,\mu g\,ml^{-1}$. On both cell lines $50\,\mu g\,ml^{-1}$ M or BCNU caused very similar inhibition of cell growth and [$^3$H]-TdR incorporation and these effects were in fact stronger after 72 h recovery time.

The effects of M and BCNU on the cell cycle of M14 and SW626 were assessed by the flow cytometric technique (Figures 3 and 4). Both drugs caused an accumulation of cells in premitotic phase. This effect was only slight at $1\,\mu g\,ml^{-1}$, clear at $10\,\mu g\,ml^{-1}$ and very marked at $50\,\mu g\,ml^{-1}$. At the

**Figure 3** Effects of 24 h M and BCNU treatment at concentrations of 1, 10 and $50\,\mu g\,ml^{-1}$ on cell cycle phase distribution in M14 human melanoma cell line.

**Figure 4** Effects of 24 h M and BCNU treatment at concentrations of 1, 10 and 50 $\mu$g ml$^{-1}$ on cell cycle phase distribution in SW626 human ovarian cancer cell line.

highest concentration M induced an accumulation of cells in SM and SLG2M, whereas BCNU caused an arrest only in SLG2M.

The activity of M was tested on 16 primary cultures of human ovarian cancer cells from 13 patients. Tables II and III show the results of flow cytometry analysis in ovarian cancer cells, derived from previously untreated or treated patients, after 24 h M treatment. When possible [$^3$H]-TdR incorporation after 24 h drug treatment was also evaluated. Only in case no. 3 was the cell cycle perturbed, with an accumulation of cells in the SE and SM phases. The slow progression through the S phase was consistent with the marked reduction of [$^3$H]-TdR incorporation observed in this case ($P<0.01$ at 1 and 10 $\mu$g ml$^{-1}$ M concentrations).

**Table II** Effects of M on the distribution in G1, S early (SE), S middle (SM), and S late +G2+Mitosis (SLG2M) and on [$^3$H]-TdR incorporation of human ovarian cancer cells (growing in primary culture) derived from patients previously untreated with antineoplastic agents. Cells were treated for 24 h.

| *Pt. no.* | *Sample* | *G1* | *SE* | *SM* | *SLG2M* | *Mitozolomide* $\mu g\,ml^{-1}$ | *DNA index* | [$^3$*H*]-*TdR* % *of control* |
|---|---|---|---|---|---|---|---|---|
| 1 | Primary tumour | 57 | 12 | 12 | 19 | control | 1.30 | |
| | | 56 | 13 | 11 | 20 | 0.1 | | 100 |
| | | 45 | 18 | 12 | 25 | 1 | | 100 |
| | | 44 | 19 | 12 | 25 | 10 | | 90 |
| 2 | Ascitic fluid | 64 | 4 | 7 | 25 | control | 1.20 | |
| | | 78 | 44 | 3 | 15 | 0.1 | | 85 |
| | | 74 | 3 | 4 | 19 | 1 | | 84 |
| | | 70 | 4 | 5 | 21 | 10 | | 70[a] |
| 3 | Metastasis | 42 | 10 | 10 | 38 | control | 1.87 | |
| | | 35 | 18 | 21 | 25 | 1 | | 37[b] |
| | | 38 | 18 | 23 | 21 | 10 | | 28[b] |
| 4 | Primary tumour | 69 | 5 | 5 | 21 | control | 1.27 | — |
| | | 71 | 5 | 6 | 18 | 1 | | |
| | | 68 | 5 | 5 | 22 | 10 | | |
| 5 | Primary tumour | 55 | 12 | 11 | 22 | control | 1.47 | — |
| | | 53 | 14 | 12 | 21 | 1 | | |
| | | 48 | 17 | 12 | 23 | 10 | | |
| 6 | Primary tumour | 71 | 8 | 8 | 13 | control | 1.80 | — |
| | | 71 | 8 | 8 | 11 | 1 | | |
| | | 72 | 7 | 9 | 12 | 10 | | |
| 6a | Ascitic fluid | 61 | 10 | 13 | 16 | control | 1.80 | — |
| | | 58 | 9 | 15 | 18 | 0.1 | | |
| | | 54 | 15 | 15 | 16 | 1 | | |
| | | 57 | 10 | 13 | 20 | 10 | | |
| 7 | Primary tumour | 36 | 18 | 16 | 30 | control | 1.00 | |
| | | 42 | 16 | 16 | 26 | 1 | | |
| | | 34 | 19 | 16 | 31 | 10 | | |

DNA index was determined as described in **Materials and methods.** Statistical analysis was by Dunnett's test. [a]$P<0.05$; [b]$P<0.01$.

### Discussion

As previously reported on murine Lewis lung carcinoma *in vivo* (Broggini *et al.*, 1986) and *in vitro* (Horgan *et al.*, 1983) M causes an accumulation of cells in the S late+G2M phase of the cell cycle.

At a high concentration M, unlike BCNU, also produces an accumulation of cells in SM phase. This may be related to quantitative or qualitative differences in the binding of the two drugs to DNA and possibly to differences in DNA damage and repair. That the biochemical features of M and BCNU differ is not suprising considering that BCNU causes protein carbamylation whereas M does not (Hickman *et al.*, 1985). It is known that the formation of DNA-ISC after chloroethylnitrosoureas treatment involves a first rapid alkylation of O$^6$guanine (i.e. chloroethylation or hydroxyethylation) followed by a second reaction with a cytosine located on the opposite strand of DNA (Erickson *et al.*, 1980). If a cell synthesized O$^6$guanine alkyl transferase (MER$^+$ phenotype) the monoadducts on O$^6$guanine are removed, thus preventing the formation of DNA-ISC. In contrast, in a cell which is O$^6$guanine repair-deficient (MER$^-$ phenotype) the monoadduct is not removed and can form DNA-ISC. Both chloroethylnitrosoureas and M induced DNA-ISC appear related to their cytotoxicity (Gibson *et al.*, 1984*b*).

However some differences which may perhaps underlie the different perturbation in the cell cycle phases were suggested by Gibson *et al.* (1984*a, b*). They found that M formed DNA-ISC more slowly than chloroethylnitrosoureas. In addition M caused much greater differential cytotoxicity between O$^6$alkylguanine repair proficient (MER$^+$ phenotype) and deficient (MER$^-$ phenotype) cells, suggesting that it forms more O$^6$guanine adducts than BCNU.

Even though these differences are of biochemical

**Table III** Effects of M on the distribution in G1, S early (SE), S middle (SM), and S late +G2+Mitosis (SLG2M) and on [$^3$H]-TdR incorporation of human ovarian cancer cells (growing in primary culture) derived from patients previously treated with antineoplastic agents. Cells were treated for 24 h.

| *Pt. no.* | *Sample* | *G1* | *SE* | *SM* | *SLG2M* | *Mitozolomide* $\mu g\,ml^{-1}$ | *DNA index* | [$^3$*H*]-*TdR* % *of control* |
|---|---|---|---|---|---|---|---|---|
| 8 | Ascitic fluid | 52 | 12 | 10 | 26 | control | 1.79 | |
| | | 55 | 10 | 11 | 24 | 0.1 | | 100 |
| | | 53 | 10 | 11 | 26 | 1 | | 100 |
| | | 47 | 10 | 11 | 32 | 10 | | 75[a] |
| 9 | Ascitic fluid | 85 | 3 | 3 | 9 | control | 1.00 | |
| | | 82 | 3 | 4 | 11 | 0.1 | | 100 |
| | | 84 | 4 | 3 | 9 | 1 | | 100 |
| | | 83 | 4 | 4 | 9 | 10 | | 100 |
| 9a | Ascitic fluid | 36 | 14 | 14 | 36 | control | 1.00 | |
| | | 43 | 14 | 16 | 27 | 0.1 | | 90 |
| | | 36 | 15 | 17 | 32 | 1 | | 82 |
| | | 34 | 14 | 16 | 36 | 10 | | 82 |
| 10 | Ascitic fluid | 35 | 13 | 20 | 22 | control | 1.57 | |
| | | 30 | 14 | 22 | 34 | 0.1 | | 100 |
| | | 29 | 16 | 22 | 33 | 1 | | 100 |
| | | 28 | 22 | 27 | 23 | 10 | | 100 |
| 11 | Primary tumour | 86 | 1 | 1 | 12 | control | 1.00 | |
| | | 88 | 1 | 1 | 10 | 1 | | 100 |
| | | 83 | 2 | 1 | 14 | 10 | | 76 |
| 11a | Metastasis | 66 | 3 | 4 | 27 | control | 1.00 | — |
| | | 66 | 2 | 4 | 28 | 1 | | |
| | | 65 | 2 | 4 | 29 | 10 | | |
| 12 | Ascitic fluid | 67 | 7 | 7 | 20 | control | 0.90 | — |
| | | 40 | 9 | 10 | 41 | 10 | | |
| 13 | Ascitic fluid | 56 | 14 | 12 | 18 | control | 1.67 | — |
| | | 55 | 14 | 13 | 18 | 0.1 | | |
| | | 55 | 13 | 12 | 20 | 1 | | |
| | | 51 | 15 | 15 | 19 | 10 | | |

DNA index was determined as described in **Materials and methods.** Statistical analysis was by Dunnett's test. [a]$P<0.05$; [b]$P<0.01$.

interest, their relevance to possible contrasting pharmacological effects is questionable since in rodent tumours M and chloroethylnitrosoureas showed a similar spectrum of activity (Hickman *et al.*, 1985) and were cross resistant (Gibson, 1982). In the present studies the cytotoxicity of M and BCNU against M14 and SW626 human cell lines was also similar.

The indication arising during phase I clinical trials that M might be active against human ovarian carcinoma (Newlands *et al.*, 1985) prompted us to investigate whether human ovarian epithelial cancer cells from biopsies of primary tumours or metastases or from ascitic fluids were sensitive to this agent. Using flow cytometry analysis, a method with proven sensitivity in detecting antiproliferative properties of this drug, we assessed whether M caused cell cycle perturbation on 16 primary cultures of ovarian carcinoma from 13 patients. M was only effective in one case in which a moderate accumulation of cells in S phase was seen. We did not test drug concentrations higher than 10 $\mu g\,ml^{-1}$ since a plasma peak level of 7 $\mu g\,ml^{-1}$ M was reported after the dose of 250 $mg\,kg^{-1}$, i.e. higher than that recommended as safe for further clinical investigations (Newlands *et al.*, 1985).

Since none of the patients had previously received drugs related to M (i.e. triazenes or nitrosoureas) it may be excluded that resistance was induced by previous treatment, and it appears more likely that ovarian carcinoma cells are naturally non-susceptible to this drug.

Whether this lack of sensitivity is due to these cells' ability to remove O$^6$alkylguanine adducts from DNA or to other mechanisms is now being investigated in our laboratories.

This work was partially supported by CNR (Progetto Oncologia No. 85.02209.44). The generous contribution of the Italian Association for Cancer Research, Milan, Italy, is gratefully acknowledged.

**References**

BROGGINI, M., ERBA, E., MORASCA, L., HORGAN, C. & D'INCALCI, M. (1986). In vivo studies with the novel anticancer agent mitozolomide (NSC 353451) on Lewis lung carcinoma. *Cancer Chemother. Pharmacol.*, **16**, 125.

COLOMBO, T., BROGGINI, M., VAGHI, M., AMATO, G., ERBA, E. & D'INCALCI, M. (1986). Comparison between VP16 and VM26 in Lewis lung carcinoma of the mouse. *Eur. J. Cancer Clin. Oncol.*, **22**, 173.

ERBA, E., VAGHI, M., PEPE, S. & 6 others (1985). DNA index of ovarian carcinomas from 56 patients: In vivo studies. *Br. J. Cancer*, **52**, 565.

ERICKSON, L.C., BRADLEY, M.O., DUCORE, J.M., EWIG, R.A. & KOHN, K.W. (1980). DNA crosslinking and cytotoxicity in normal and transformed human cells treated with antitumor nitrosoureas. *Proc. Natl Acad. Sci. USA*, **77**, 467.

FOGH, J., FOGH, J.M. & ORFEO, T. (1977*a*). One hundred and twenty-seven cultured human tumor cell lines producing tumors in nude mice. *J. Natl Cancer Inst.*, **59**, 221.

FOGH, J., WRIGHT, W.C. & LOVELESS, J.D. (1977*b*). Absence of HeLa cell contamination in 169 cell lines derived from human tumors. *J. Natl Cancer Inst.*, **58**, 209.

GIBSON, N.W. (1982). *Investigation into the Mechanism of Action of Antineoplastic Nitrosoureas*. PhD Dissertation, University of Aston in Birmingham.

GIBSON, N.W., ERICKSON, L.C. & HICKMAN, J.A. (1984*a*). Effects of the antitumor agent 8-carbamoyl-3(2-chloroethyl)imidazo[5, 1-d]-1, 2, 3, 5,-tetrazin-4(3H)-one on the DNA of mouse L1210 cells. *Cancer Res.*, **44**, 1767.

GIBSON, N.W., HICKMAN, J.A. & ERICKSON, L.C. (1984*b*). DNA cross-linking and cytotoxicity in normal and transformed human cells treated in vitro with 8-carbamoyl-3(2-chloroethyl)imidazo[5, 1-d]-1, 2, 3, 5-tetrazin-4(3H)-one. *Cancer Res.*, **44**, 1772.

GOLUB, S.H., HANSON, D.C., SULIT, H.L., MORTON, D.L., PELLEGRINO, M.A. & FERRONE, S. (1976). Comparison of histocompatibility antigens on cultured human tumor cells and fibroblasts by quantitative antibody absorption and sensitivity to cell-mediated cytotoxicity. *J. Natl Cancer Inst.*, **56**, 167.

HICKMAN, J.A., STEVENS, M.F.G., GIBSON, N.W. & 6 others (1985). Experimental antitumor activity against murine tumor model systems of 8-carbamoyl-3-(2-chloroethylimidazo[5, 1-d]-1, 2, 3, 5-tetrazin-4(3H-one (mitozolomide), a novel broad-spectrum agent. *Cancer Res.*, **45**, 3008.

HORGAN, C.M.T. & TISDALE, M.J. (1984). Antitumour imidazotetrazines. IV. An investigation into the mechanism of antitumour activity of a novel and potent antitumour agent, mitozolomide (CCRG 81010, M & B 39565; NSC 353451). *Biochem Pharmacol.*, **33**, 2185.

HORGAN, C.M.T., TISDALE, M.J., ERBA, E., D'INCALCI, M. & PEPE, S. (1983). Flow cytometric analysis of DNA distribution in Lewis lung carcinoma cells after treatment with CCRG 81010 (M & B 39565). *Br. J. Cancer*, **48**, 139.

KRISHAN, A. & FREI, E. III (1976). Effect of adriamycin on the cell cycle traverse and kinetics of cultured human lymphoblasts. *Cancer Res.*, **36**, 143.

MORASCA, L., ERBA, E., VAGHI, M. & 5 others (1983). Clinical correlates of in vitro drug sensitivities of ovarian cancer cells. *Br. J. Cancer*, **48**, 61.

NEWLANDS, E.S., BLACKLEDGE, G. SLACK, J.A. & 4 others (1985). Phase I clinical trial of mitozolomide. *Cancer Treat. Rep.*, **69**, 801.

STEVENS, M.F.G., HICKMAN, J.A., STONE, R. & 4 others (1984). Antitumor imidazotetrazines. 1. Synthesis and chemistry of 8-carbamoyl-3-(2-cloroethyl) imidazo [5,1-d]-1,2,3,5-tetrazin-4(3H)-one, a novel broad-spectrum antitumor agent. *J. Med. Chem.*, **27**, 196.